# A Treatise on Protracted Indigestion and Its Consequences; Being the Application to the Practical Department of Medicine of the Results of an Inquiry into the Laws of the Vital Functions. &c. &c.

**Published:** 1843-08-05

**Authors:** 


*A Treatise on Protracted Indigestion and its Consequences; being the application to the Practical Department of Medicine of the Results of an Inquiry into the Laws of the Vital Functions: Addressed by the Author on his Retirement from the Medical Profession, both to the Members of that Profession, and to the well-educated Public, particularly Parents.* By A. P. W. Philip, M. D., F. R. S. London and Edinburgh, &c. &c. Philadelphia: Lea & Blanchard, 1843. 8vo. pp. 240.

This Valedictory Address of Dr. Wilson Philip is addressed chiefly to the public, for what reason we cannot conceive, unless the author shared our conviction that but few of his medical brethren would be tempted to read it, and still fewer would be able to comprehend it. The work is a *rechauffé* of old errors, and exploded notions very clumsily thrown together, with an utter contempt of any thing like order or method. The author's complacency is in the highest degree amusing, and the manner in which he attempts to correct the "physiological errors" of the profession displays a most disinterested spirit. Dr. Philip's ingenuousness does not allow him, however, to mention the fate of his own physiological investigations and their results. He makes no mention of their refutation by Müller, Diekhof, Breschet and Edwards, and the entire disproval of the hasty theories he so unphilosophically framed, and which he reiterates *pro bono publico* in the present publication. All fellow labourers in physiological inquiry our author never deigns to mention, doubtless from the fear of perplexing the minds of those for whom the work is especially destined. The treatise on Indigestion is served up in the second part, somewhat altered, but little amended. Eulogies on minute doses of calomel, and the tonic properties of kreosote are the staple of this part of the volume, with a section or two on distended liver, a disease which we are disposed to consider as the last pathological novelty, and which we almost fear our dulness will not permit us to recognise through Dr. Philip's description.

Nearly one-half of the volume is occupied by a republication of the author's memoirs presented to the Royal Society.

